# Development of a natural language processing algorithm to detect chronic cough in electronic health records

**DOI:** 10.1186/s12890-022-02035-6

**Published:** 2022-06-28

**Authors:** Vishal Bali, Jessica Weaver, Vladimir Turzhitsky, Jonathan Schelfhout, Misti L. Paudel, Erin Hulbert, Jesse Peterson-Brandt, Anne-Marie Guerra Currie, Dylan Bakka

**Affiliations:** 1 Center for Observational and Real-World Evidence (CORE), Merck & Co., Inc., Rahway, NJ USA; 2 Health Economics and Outcomes Research (HEOR), Optum Insight, Eden Prairie, MN USA; 3 Optum Enterprise Analytics (OEA), Optum Insight, Eden Prairie, MN USA; 4 Present Address: Henry M. Jackson Foundation for the Advancement of Military Medicine, Bethesda, MD USA

**Keywords:** Cough, Chronic cough, Natural language processing, Electronic health records, Sensitivity and specificity, Diagnostic test accuracy study

## Abstract

**Background:**

Chronic cough (CC) is difficult to identify in electronic health records (EHRs) due to the lack of specific diagnostic codes. We developed a natural language processing (NLP) model to identify cough in free-text provider notes in EHRs from multiple health care providers with the objective of using the model in a rules-based CC algorithm to identify individuals with CC from EHRs and to describe the demographic and clinical characteristics of individuals with CC.

**Methods:**

This was a retrospective observational study of enrollees in Optum’s Integrated Clinical + Claims Database. Participants were 18–85 years of age with medical and pharmacy health insurance coverage between January 2016 and March 2017. A labeled reference standard data set was constructed by manually annotating 1000 randomly selected provider notes from the EHRs of enrollees with ≥ 1 cough mention. An NLP model was developed to extract positive or negated cough contexts. NLP, cough diagnosis and medications identified cough encounters. Patients with ≥ 3 encounters spanning at least 56 days within 120 days were defined as having CC.

**Results:**

The positive predictive value and sensitivity of the NLP algorithm were 0.96 and 0.68, respectively, for positive cough contexts, and 0.96 and 0.84, respectively, for negated cough contexts. Among the 4818 individuals identified as having CC, 37% were identified using NLP-identified cough mentions in provider notes alone, 16% by diagnosis codes and/or written medication orders, and 47% through a combination of provider notes and diagnosis codes/medications. Chronic cough patients were, on average, 61.0 years and 67.0% were female. The most prevalent comorbidities were respiratory infections (75%) and other lower respiratory disease (82%).

**Conclusions:**

Our EHR-based algorithm integrating NLP methodology with structured fields was able to identify a CC population. Machine learning based approaches can therefore aid in patient selection for future CC research studies.

**Supplementary Information:**

The online version contains supplementary material available at 10.1186/s12890-022-02035-6.

## Background

Chronic cough (CC)—defined as daily cough for ≥ 8 weeks [1, 2]—is associated with poor health-related quality of life [3–8]; sleep, work, and activity impairment [3, 6, 7]; and high rates of health care resource use (HCRU) [6, 7, 9–11]. Many cases are related to underlying medical conditions, such as asthma, upper airway cough syndrome (formerly postnasal drip), and gastro-esophageal reflux disease; smoking or vaping, or prescription drugs [10, 11]; and often resolve upon treatment of the underlying cause. However, a subset of patients experiences unexplained and/or treatment-refractory CC [12–15]. Current clinical guidelines for treating such patients recommend trying a series of medications; however, most of the people with CC report insufficient relief from their treatment [16, 17].

Lack of a specific diagnostic code for CC precludes identification of representative patient populations from health records. The International Classification of Diseases (ICD) code for cough is used inconsistently, and misdiagnosis is common [15]. Most previous studies have typically relied on general population survey approaches to identify individuals with CC, or focused on the subset of individuals referred to specialist cough clinics. In sample populations identified by these two methods, CC has been reported to predominantly affect women, with a peak incidence at > 50 years of age [3, 7, 18–20].

Future CC research and treatment will benefit from new methods to accurately identify representative patient populations. Natural language processing (NLP) approaches to extracting mentions of cough from provider notes in electronic health records (EHRs) offer an alternative identification method. We and others have reported NLP algorithms that identified cough mentions from EHRs with a positive predictive value (PPV) of ~ 97% [17, 20]. Weiner et al. identified 23,371 individuals with CC using an algorithm that combined NLP-defined cough mentions with structured data on medications and diagnosis codes [17]. In our previous study we developed an NLP algorithm using EHRs from a single provider’s administrative database [20]. The study population included individuals with specialist-diagnosed CC, indicated by an internal CC encounter code. The NLP algorithm identified additional individuals with predicted CC from the same database; this clinically undiagnosed group had lower rates of diagnostic testing and medication prescriptions than the specialist-diagnosed CC group, and higher HCRU [20]. These findings illustrate the importance of enhanced methods to identify and characterize CC, to improve its diagnosis and treatment.

The objectives of this study were to develop and validate an NLP algorithm to identify cough in free-text provider notes in EHRs in a more heterogeneous data set drawn from multiple payer and provider networks; to use it to identify individuals with CC from their EHRs; and to describe the demographic, clinical, and HCRU characteristics of individuals with CC. High precision/PPV was prioritized over recall/sensitivity.

### Methods

#### Study design

This was a retrospective observational study of EHRs from administrative databases. All data were accessed in compliance with the United States Health Insurance Portability and Accountability Act, and no identifiable protected health information was extracted. All databases used are statistically certified as de-identified and no electronic or paper copies of medical charts were available. Informed consent and Institutional Review Board approval were not required.

#### Study sample

The study population included individuals registered in Optum’s Integrated Clinical + Claims Database who had overlapping periods of enrollment in the Optum Research Database and the Optum Clinical Database from January 2016 through March 2019. The integrated database combines claims information with EHRs and includes records from ~ 60 provider delivery organizations in the United States and Puerto Rico. An average of 45 months of data is included for each of > 101 million unique individuals, who are enrollees in national commercial health plans or Medicare Advantage with Part D (MAPD). These data include details of physician office visits and hospital stays; medications, procedures, and diagnoses; and information derived from physician, radiology, and pathology reports using proprietary NLP methods.

The study inclusion criteria required enrollment in a commercial or MAPD plan in the Optum Research Database, with an identification period between January 1, 2016 and March 31, 2017 (Additional file 1: Figure S1). The earliest observed date of enrollment within these dates was defined as the index date. Eligible participants were 18–85 years of age in the index year. Continuous health plan enrollment, including medical and pharmacy coverage, was required for ≥ 24 months after or including the index date. Participants required a patient ID in the Optum Research Clinical Database, with a clinical activity period (the period between the earliest and latest observed active dates) that overlapped with the study identification period. An overlapping observable period of ≥ 24 months in the Optum Research Database and Optum Research Clinical Database was required. Patients were excluded if there was evidence of ACE inhibitor use (written medication order or prescription fill claim) during the study period.

### Development of a gold-standard data set

There is currently no gold standard for identification of cough from administrative data and it is unknown whether acute cough diagnosis codes are entered consistently. We therefore developed a labeled data set with which to train, validate, and test an NLP model. Provider notes were first reviewed to understand the general concepts and complexity of the narrative relating to cough. Semi-automated pre-annotation was then conducted using Webanno, a general-purpose tool for the annotation of linguistic structures in free text [21]. Linguistic cough terms for this pre-annotation phase were included based on preliminary assessment of their frequency and co-appearance. The terms used included cough, coughed, coughing, coughs, cougher, and tussis. Linguistic terms for expectorate were not included after a preliminary analysis showed that they rarely occurred in the absence of a linguistic term for cough.

Patients with ≥ 1 mention of a linguistic term for cough in any provider note from January 2016 to March 2019 were identified. One thousand documents among the provider notes of these patients were then randomly selected for manual annotation. Each document was annotated by two independent annotators employed by Optum. A third annotator curated the data and resolved any conflicts. Each annotator had a background in medicine, clinical research, or health informatics, and clinical proficiency in medical chart review and abstraction. Inter-annotator agreement was calculated using Krippendorf’s Alpha [22]. The final annotated corpus was split into a training set (600 documents), validation set (200), and test set (200).

### Development of an EHR-based NLP cough algorithm

The training set of the annotated corpus was used to develop an NLP algorithm for cough. The NLP algorithm used supervised machine learning techniques, including discriminative sequence labeling models (e.g., Conditional Random Field) and classifiers (e.g., Support Vector Machine, Random Forest) to extract positive mentions of cough (entity-recognition) and contextual qualifiers for each mention (classification); examples are provided in Additional file 1: Table S1. Contextual qualifiers were also extracted for each mention. For example, text such as ‘medication may cause cough’ indicates a hypothetical cough situation, whereas ‘patient denies having a cough’ indicates negative cough. Further examples are provided in Additional file 1: Table S2. In the final NLP model, cough terms were classified and labeled as positive cough contexts if an approved cough term was present and extracted, cough was relevant to the patient, and the cough context was not negated.

During algorithm development, we performed an exploratory analysis of the temporality of cough mentions, to understand whether the cough was new or ongoing. True historical cough mentions were rare; in almost every case where a historical term was used, the cough was determined to be a current problem. For example, provider notes that mentioned temporality included ‘Current smoker with history of cough × 1 month’ and ‘Patient with history of cough × 5 weeks improving’. We therefore did not include any measure of temporality in the cough algorithm.

The test data set was used to evaluate the performance of the NLP cough model in extracting cough contexts and classifying positive and negative cough contexts against the gold standard annotated corpus. The success criterion was set at a precision/PPV value of > 0.90 based on the gold standard held-out test set.

### Development of an EHR-based chronic cough algorithm

The date of each unique positive NLP cough mention was recorded. Outpatient cough encounters were identified from EHRs, from ambulatory interactions (office or clinic visits), day surgery, home visits, and urgent care encounters that included a diagnosis code for cough (ICD-10 R05). We also recorded medication orders in the EHR data; relevant cough medications contained benzonatate or dextromethorphan. Expectorants containing guaifenesin, other mucoactive agents, and codeine agents were excluded as these medications are not specific to cough. The dates of outpatient encounters and medication orders were recorded, with a maximum of one per category per day for each individual.

The CC algorithm defined CC as ≥ 3 positive cough encounters within 120 days, with ≥ 56 days between the first and last qualifying encounter. Any combination of three encounter types (NLP cough mentions, diagnosis codes, written medication orders) within the qualifying period was included. The date of the third qualifying encounter (the eligibility date) was recorded.

A sensitivity test was performed by supplementing cough encounters identified from EHRs with encounters identified from claims data. The dates of all claims for prescription fills for cough medications (as above), as well as cough diagnosis codes (ICD-10 R05) in outpatient claims, were recorded, with a maximum of one per person per day. The CC algorithm applied to this expanded set of cough encounter data used the same rules outlined above.

### Characteristics of patients with chronic cough

Socio-demographic information was obtained from EHRs. Comorbidities during the 24-month observation period were defined using the Agency for Healthcare Research and Quality’s Clinical Classifications Software [23]. All-cause health care costs were obtained from claims data and computed as averaged per patient per month (PPPM) values during the 24-month observation period. Costs included the combined health plan and patient paid amounts and were labeled as pharmacy or medical, with the latter category comprising inpatient, emergency room, and ambulatory care. Costs were adjusted using the annual medical care component of the United States consumer price index to reflect inflation between 2016 and 2018 [24]. To assess all-cause HCRU, binary indicators and counts of all-cause ambulatory visits, emergency room visits, inpatient admissions, and pharmacy claims were computed for the full 24-month observation period and presented as PPPM values.

### Analysis

True positive (TP), false positive (FP), true negative (TN), and false negative (FN) results were used in the calculation of study metrics. Sensitivity /recall was defined as the TP rate, or the proportion of positive or negative cough mentions correctly identified by the NLP model: TP/(TP + FN). Precision / PPV was defined as the proportion of patients labeled as positive or negative for cough by the NLP model that were correctly identified: TP/(TP + FP). F1, the harmonic mean of precision and recall, was computed as (2 × (precision × recall)/(precision + recall)).

Descriptive statistics were used to quantify the prevalence of CC and the demographic characteristics of patients with CC.

## Results

### Participants

The final sample comprised 327,423 individuals (Additional file 1: Figure S2).

### Natural language processing cough model

Manual annotation of 1000 documents randomly selected from among the records of individuals with ≥ 1 cough mention identified 1718 individual cough contexts. Inter-annotator agreement was calculated with an α of 0.89.

The performance of the final NLP algorithm on the held-out test data set is summarized in Table 1. The precision of the NLP model for extracting cough contexts was 0.98, and the precision for classifying positive and negative cough mentions was 0.96 in both cases. Recall for extracting cough mentions was 0.98; recall for positive and negative cough contexts was 0.68 and 0.84, respectively. The F1 scores were 0.98 for extracting cough contexts, 0.80 for classifying positive cough mentions, and 0.89 for classifying negative cough mentions.

**Table 1:** Cough model performance metrics on the held-out test set

Concept	Model type	Precision (PPV)*	Recall (Sensitivity)^†^	F1^‡^	Support^§^
Cough mention	Entity recognition	0.98	0.99	0.99	341
Positive cough mention	Classification	0.96	0.68	0.80	179
Negative cough mention	Classification	0.96	0.84	0.89	86

Positive Predictive Value (PPV)

*Precision (PPV) indicates how well the model correctly identifies the desired cough context. PPV = true positives/(true positives + false positives)

^†^Recall (Sensitivity) indicates how well true positive cough contexts have been captured from provider notes. Recall = true positives/(true positives + false negatives)

^‡^F1 represents the harmonic mean of precision and recall and identifies the relative impacts of false positives and false negatives on the interpretation of the model’s performance. F1 = (2 × (precision × recall)/(precision + recall))

^§^Support represents the number of occurrences of the cough concept in the test set

### EHR-based chronic cough algorithm

**Fig. 1:**
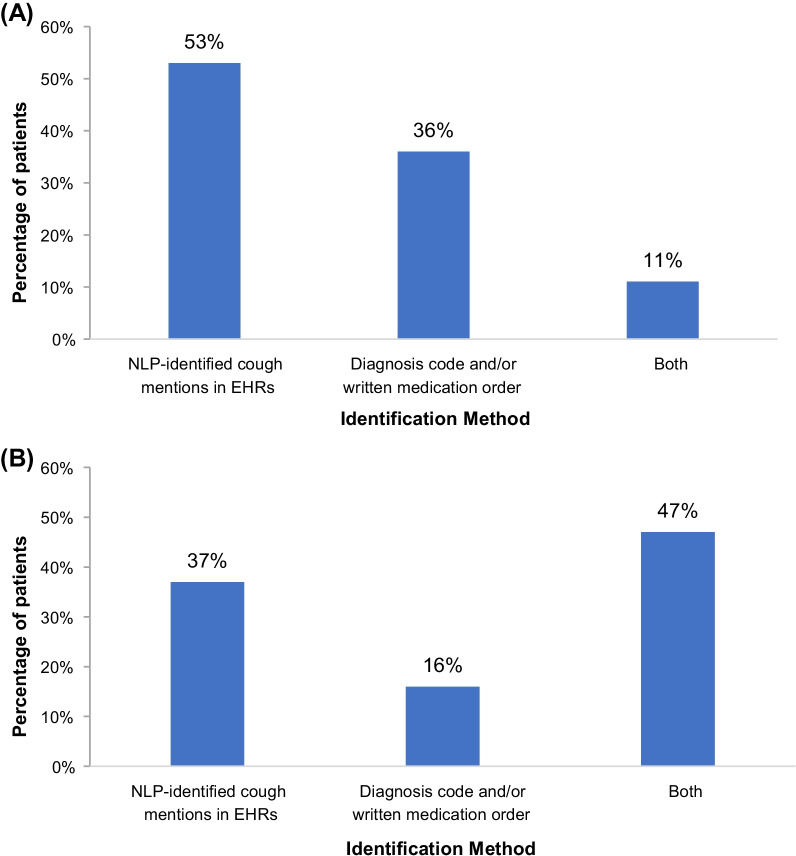
Proportion of **A** cough encounters and **B** individuals with chronic cough identified from electronic health records, diagnosis codes/written medication orders, or a combination of both methods. EHR, electronic health record; NLP, natural language processing. Panel (**A**), individuals with cough encounters (*N* = 291,326). Panel (**B**), individuals with chronic cough (*N* = 8861)

The algorithm identified 291,326 individual cough encounters from the full EHRs of 327,423 plan enrollees; 128,467 individuals had ≥ 1 cough encounter during the observation period. A majority (53%) of these encounters were identified via NLP positive cough mentions in provider notes alone, while 36% were identified through diagnosis codes and/or written medication orders, and 11% through a combination (Fig. 1A).

Chronic cough (≥ 3 cough mentions within a 120-day period, with ≥ 56 days between the first and last qualifying record) was identified in 4818 patients (1.5% of the sample). Thirty-seven percent of this group were qualified using NLP-identified positive cough mentions in provider notes alone, 16% by diagnosis codes and/or written medication orders alone, and 47% through a combination of both provider notes and diagnosis codes/medications (Fig. 1B). Among patients identified as having CC, the mean number of cough encounters per patient during the 24-month observation period was 8.9 (range 3–147).

We performed a sensitivity analysis by supplementing the EHR-based CC algorithm with available claims data for prescription fills for cough medications and cough diagnosis codes during the 24-month observation period. Among 327,423 individual enrollees, 250,889 had ≥ 1 cough encounter, with a total of 571,223 cough encounters. Using this larger set of encounters, the expanded algorithm identified 8861 individuals with CC (2.7% of the sample).

### Characteristics of individuals with chronic cough

The 4818 individuals identified as having CC from their EHRs had a mean (standard deviation, SD) age of 61.0 (15.3) years. Additional demographic characteristics are summarized in Table 2. Two-thirds the CC individuals were female, and almost half were ≥ 65 years of age. The most prevalent comorbidities were other lower respiratory disease (81.5%), respiratory infections (74.6%), disorders of lipid metabolism (62.2%), other connective tissue disease (61.7%), and diseases of the heart (58.6%; Table 2).

**Table 2:** Demographic characteristics and comorbidities of individuals with chronic cough

Characteristic	Individuals with chronic cough (*N* = 4818) N (%)
*Age group (years)*	
18–39	508 (10.5)
40–44	207 (4.3)
45–49	324 (6.7)
50–54	432 (9.0)
55–59	505 (10.5)
60–64	501 (10.4)
≥ 65	2341 (48.6)
*Sex*	
Female	3215 (66.7)
Region*	
Northeast	677 (14.1)
Midwest	2297 (47.7)
South	1362 (28.3)
West	480 (10.0)
Other	2 (0.0)
*Comorbidities*^†^	
Other lower respiratory disease	3926 (81.5)
Respiratory infections	3595 (74.6)
Disorders of lipid metabolism	2996 (62.2)
Other connective tissue disease	2974 (61.7)
Diseases of the heart	2824 (58.6)
Hypertension	2808 (58.3)
Non-traumatic joint disorders	2774 (57.6)
Eye disorders	2687 (55.8)
Other nutritional, endocrine, and metabolic disorders	2550 (52.9)
Other skin disorders	2534 (52.6)

*Northeast: Connecticut, Maine, Massachusetts, New Hampshire, New Jersey, New York, Pennsylvania, Rhode Island, Vermont. Midwest: Illinois, Indiana, Iowa, Kansas, Michigan, Minnesota, Missouri, Nebraska, North Dakota, Ohio, South Dakota, Wisconsin. South: Alabama, Arkansas, Delaware, District of Columbia, Florida, Georgia, Kentucky, Louisiana, Maryland, Mississippi, North Carolina, Oklahoma, South Carolina, Tennessee, Texas, Virginia, West Virginia. West: Alaska, Arizona, California, Colorado, Hawaii, Idaho, Montana, Nevada, New Mexico, Oregon, Utah, Washington, Wyoming. Other: American Samoa, Armed Forces, Commonwealth of the Northern Mariana Islands, Federated State of Micronesia, Guam, Marshall Islands, Palau, Puerto Rico, Virgin Islands

^†^Comorbidities were as defined by the Agency for Healthcare Research and Quality’s Clinical Classifications Software Refined (CCSR) for ICD-10-CM Diagnoses [23].

Overall, 96.9% of individuals with CC had ≥ 1 ambulatory health care visit over the 24-month observation period, 96.4% used a pharmacy service, 58.3% had ≥ 1 emergency room visit, and 27.9% had ≥ 1 inpatient stay (Table 3). The mean (SD) PPPM HCRU during the study period was 3.18 (2.97) pharmacy fills, 2.37 (2.31) ambulatory visits, 0.12 (0.26) emergency room visits, and 0.02 (0.05) inpatient stays, with a mean (SD) inpatient stay of 18.66 (29.63) days. The mean (SD) total health care cost was $1931 ($4281) PPPM, for an average annual cost of $23,172 per patient (Table 4). Medical costs were higher than pharmacy costs ($1450 [3749] versus $481 [$1440] PPPM), with ambulatory visits making the largest contributions to the former category at $771 ($1813) PPPM.

**Table 3:** All-cause health care resource use among individuals with chronic cough

All-cause HCRU	Individuals with chronic cough (*N* = 4818) *N* (%)	HCRU counts, PPPM Mean (SD)
Ambulatory visit	4666 (96.9)	2.37 (2.31)
Office visit	4624 (96.0)	1.29 (1.12)
Hospital outpatient visit	4281 (88.9)	1.09 (1.85)
Emergency room visit	2811 (58.3)	0.12 (0.26)
Hospital inpatient visit	1342 (27.9)	0.02 (0.05)
Pharmacy	4644 (96.4)	3.18 (2.97)

HCRU, health care resource use; PPPM, per patient per month; SD, standard deviation

**Table 4:** All-cause health care costs for individuals with chronic cough

All-cause cost type	Individuals with chronic cough (*N* = 4818)
	Mean (SD)*
Total health care	$1931 (4281)
Medical	$1450 (3749)
Ambulatory	$771 (1813)
Office visit	$221 (448)
Hospital outpatient	$550 (1712)
Emergency room	$62 (171)
Inpatient	$468 (2804)
Other medical	$150 (764)
Pharmacy	$481 (1440)

*All values are in US dollars and represent costs per patient per month; SD, standard deviation

**Fig. 2:**
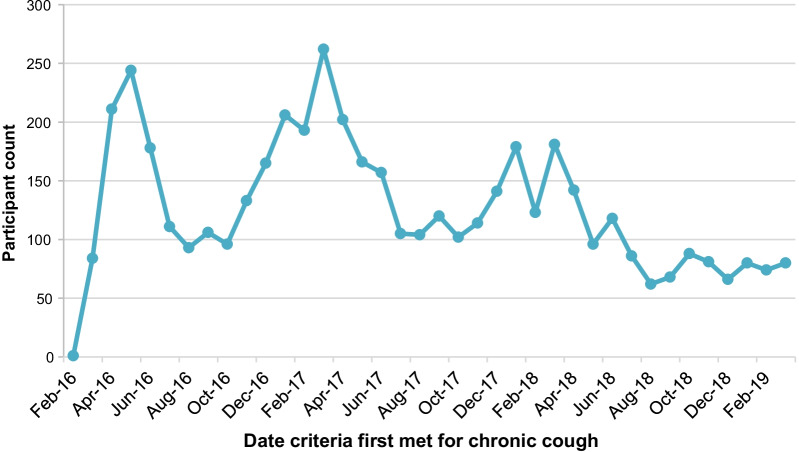
Distribution of chronic cough eligibility dates. Dates reflect the month and year in which individuals first qualified as having chronic cough

Figure 2 depicts the distribution of dates on which individuals first qualified as having CC, 56–120 days after the first qualifying cough encounter. The mean (SD) gap between first and third qualifying encounter was 67.72 (35.07) days.

## Discussion

In this study we created a rules-based algorithm to identify individuals with CC. This was achieved by developing an NLP model that can identify and classify cough contexts from provider notes in EHRs. The models were tuned to favor precision over recall. Our model had high precision—i.e., a low false positive rate—and exceeded our success criteria. The model also had high recall—i.e., a low false negative rate—for extracting cough mentions, satisfactory recall for positive cough contexts, and good recall for negative cough. The F1 score, an assessment of the relative impacts of false positives and false negatives on the interpretation of the model’s performance, was balanced for extracting cough contexts and for classifying positive cough mentions.

A chronic cough algorithm that combined free-text NLP cough model output with structured EHR data was successfully deployed to identify CC. Cough mentions identified by NLP were useful in identifying 84% of the individuals who qualified as experiencing CC using the EHR algorithm. Weiner et al. reported that 74% of individuals with CC were identified from provider notes alone and 15% from diagnosis codes and medication orders alone, compared to corresponding values of 37% and 16% for our algorithm [17]. The two studies used similar methodologies that generated comparable PPVs. These findings suggest that rules-based algorithms integrated with NLP approaches can be reliably used to identify CC with sufficient flexibility to permit their use across health care systems that use different methods to capture and record cough concepts in provider notes and diagnostic fields.

We successfully replicated and expanded on previous work to develop algorithms for cough and CC, using a more heterogeneous data set comprising information from multiple provider networks [17, 20]. Our sensitivity analysis demonstrated that integrating the outputs of the EHR-based CC algorithm with available claims data could almost double the number of individuals identified as having CC. Nevertheless, all subsequent analyses were performed using the results of the EHR-based cough model alone. Although the expanded algorithm identified additional individuals with CC, it was not possible to ascertain whether cough diagnosis codes in claims data reflected current or historical diagnoses; any carry-through from before the study’s observation period would introduce false positives.

Chronic cough, as identified by our model, was present in 1.5% of the sample population. This is lower than previous prevalence estimates of ~ 5% from general population surveys, but similar to the 1.04% prevalence reported in our previous work on NLP-mediated identification of CC from administrative data [6, 7, 20]. The generalizability of our sample population to the overall US population is unknown. The chronic cough population we identified had a mean age of 61 years and was 67% female, similar to the findings of previous NLP studies by Weiner et al. (54 years, 61% female) and Zeiger et al. (57 years, 68% female) [17, 20]. The female preponderance of CC has been consistently reported in populations identified using various methods, and may be related to sex-specific differences in cough reflex sensitivity [19, 25, 26]. Analysis of the eligibility dates on which patients qualified as having CC suggest a seasonality effect, consistent with acute respiratory infections as common etiological factors for CC, as well as previous reports that cold air is a common cough trigger [27–32].

Lower respiratory disease and respiratory infections were the most prevalent categories of comorbidity among individuals with CC. The ‘other lower respiratory disease’ category includes the ICD-10 diagnosis code for ‘cough’ (R05), which was used by the algorithm to help identify CC in some members of the sample population; this category might therefore be over-represented in our sample. The ‘respiratory infections’ category includes postnasal drip (ICD-10 code R0982), which is a common cause of CC [33, 34]. The CHEST guidelines suggest that asthma, gastroesophageal reflux disease, nonasthmatic eosinophilic bronchitis and upper airway cough syndrome are the common causes of CC. The diverse comorbidities experienced by individuals with CC in our sample reflect the heterogeneity of disease etiology in this population, highlighting the need for effective new diagnosis and treatment strategies for CC.

Individuals with CC used substantial amounts of health care resources, consistent with previous reports [3, 9, 20]. The study definition of CC required ≥ 3 ambulatory health care visits within a 120-day period, which might have selected for individuals who frequently seek medical care. The age and high comorbidity burden of our sample population might also have contributed to the high rates of HCRU we observed. Individuals with CC often undergo several rounds of diagnostic testing and specialist referrals for their condition, and many are prescribed various medications with low rates of long-term benefits [6, 7, 10, 11, 16, 20, 35, 36]. The high medical and pharmacy costs identified in the current study may reflect similar experiences with unsuccessfully seeking medical care for CC. The health care costs we calculated did not include patient-paid costs of over-the-counter medications, which are commonly used by individuals with CC [16, 35]. Thus, this study might provide conservative estimates of the economic burden of CC. Further detailed analysis of all-cause and cough-specific HCRU and associated costs in the study population is needed.

A strength of the current study is that the EHR database used aggregated comprehensive clinical and demographic data for > 100 million enrollees, from a network of 140,000 providers, and is therefore more nationally representative than any single-provider US system. Robust annotation was integrated with NLP technology to obtain clinical cough concepts from provider notes. The structure of this database is also different to that of the databases used in previous studies of NLP-based identification of CC; the success of NLP cough model in this heterogeneous data set demonstrates generalizability of this methodology.

This study has some limitations. The gold standard data set we developed to train the NLP algorithm used annotated provider notes, which might not be as accurate as chart reviews. In addition, cough encounters and diagnoses might not be recorded consistently across providers and provider networks. It is possible that some of the patients qualified as experiencing CC based on ≥ 3 cough mentions within a 56–120-day period had in fact experienced independent occurrences of acute cough. Finally, this study only validated the NLP model for capturing cough; further research is needed to validate the CC algorithm.

## Conclusions

In conclusion, a rules-based CC algorithm incorporating provider notes, diagnosis codes, and medication information was useful in identifying individuals with CC from EHRs, and in identifying characteristics of the condition such as seasonality and comorbidities. Integrating EHR-based CC algorithms with supplemental information such as claims data can identify additional CC patients, although the accuracy of such data has not yet been rigorously determined. Using NLP methods to identify individuals with CC from EHRs can facilitate new insights into the unmet needs of this population.

## Supplementary Information

**Additional file 1: Supplemental Table 1.** Examples of positive cough mentions in provider notes. **Supplemental Table 2.** Examples of negative and other non-applicable cough mentions. **Supplemental Figure 1.** Participant identification and observation periods. **Supplemental Figure 2.** Participant disposition diagram.

## Data Availability

The data that support the findings of this study are available from Optum’s Integrated Clinical + Claims Database but restrictions apply to the availability of these data, which were under license for the current study, and so are not publicly available. Individuals interested in licensing the data used for this study to conduct their own research can send their request to connected@optum.com.

## Abbreviations

CC: Chronic cough
EHRs: Electronic health records
FN: False negative
FP: False positive
HCRU: Health care resource use
ICD: International Classification of Diseases
MAPD: Medicare advantage with part D
NLP: Natural language processing
PPPM: Per patient per month
PPV: Positive predictive value
SD: Standard deviation
TN: True negative
TP: True positive

## References

[1] Irwin RS, Baumann MH, Bolser DC (2006). Diagnosis and management of cough executive summary: ACCP evidence-based clinical practice guidelines. Chest.

[2] Morice AH, Millqvist E, Bieksiene K, et al. ERS guidelines on the diagnosis and treatment of chronic cough in adults and children. Eur Respir J. 2020;55(1). 10.1183/13993003.01136-2019 PMC6942543 31515408

[3] Chamberlain SA, Garrod R, Douiri A (2015). The impact of chronic cough: a cross-sectional European survey. Lung.

[4] French CL, Irwin RS, Curley FJ, Krikorian CJ (1998). Impact of chronic cough on quality of life. Arch Intern Med.

[5] Koskela HO, Lätti AM, Purokivi MK (2017). Long-term prognosis of chronic cough: a prospective, observational cohort study. BMC Pulm Med.

[6] McGarvey L, Morice AH, Way NA, et al. Burden of chronic cough in the United Kingdom: results from the 2018 National Health and Wellness Survey (in preparation). 10.1183/23120541.00157-2023 PMC10350679 37465559

[7] Meltzer EO, Zeiger RS, Dicpinigaitis P (2021). Prevalence and burden of chronic cough in the United States. J Allergy Clin Immunol Pract.

[8] Young EC, Smith JA (2010). Quality of life in patients with chronic cough. Ther Adv Respir Dis.

[9] Koskela HO, Lätti AM, Pekkanen J. The impacts of cough: a cross-sectional study in a Finnish adult employee population. ERJ Open Res. 2018;4(4). 10.1183/23120541.00113-2018 PMC6230813 30443552

[10] Schappert SM, Nelson C (1999). National ambulatory medical care survey: 1995–96 summary. Vital Health Stat.

[11] Zeiger RS, Schatz M, Butler RK, Weaver JP, Bali V, Chen W (2020). Burden of specialist-diagnosed chronic cough in adults. J Allergy Clin Immunol Pract.

[12] McGarvey L (2013). The difficult-to-treat, therapy-resistant cough: why are current cough treatments not working and what can we do?. Pulm Pharmacol Ther.

[13] McGarvey LP (2005). Idiopathic chronic cough: a real disease or a failure of diagnosis?. Cough.

[14] Pratter MR (2006). Unexplained (idiopathic) cough: ACCP evidence-based clinical practice guidelines. Chest.

[15] Good JT, Rollins DR, Kolakowski CA, Stevens AD, Denson JL, Martin RJ (2018). New insights in the diagnosis of chronic refractory cough. Respir Med.

[16] Shelfhout J. Patient perspectives on the impact, diagnosis, and treatment of chronic cough: a descriptive analysis of United Kingdom adults (in preparation).

[17] Weiner M, Dexter PR, Heithoff K, et al. Identifying and characterizing a chronic cough cohort through electronic health records. Chest. 2020. 10.1016/j.chest.2020.12.011 33345951

[18] Mcgarvey L, Morice A, Way N (2019). Prevalence of chronic cough, patient characteristics and health outcomes among UK adults. Eur Respir J..

[19] Morice AH, Jakes AD, Faruqi S (2014). A worldwide survey of chronic cough: a manifestation of enhanced somatosensory response. Eur Respir J..

[20] Zeiger RS, Xie F, Schatz M (2020). Prevalence and characteristics of chronic cough in adults identified by administrative data. Perm J.

[21] Eckart de Castilho R, Mujdricza-Maydt E, Yimam SM, et al. A web-based tool for the integrated annotation of semantic and syntactic structures. Workshop on Language Technology Resources and Tools for Digital Humanities (LT4DH). Osaka, Japan; 2016.

[22] Krippendorff K. Computing Krippendorff's Alpha-Reliability. http://repository.upenn.edu/asc_papers/43.

[23] Healthcare Cost and Utilization Project (HCUP). Clinical Classifications Software Refined (CCSR). Clinical Classifications Software Refined (CCSR) for ICD-10-CM Diagnoses [March 5, 2021; www.hcup-us.ahrq.gov/toolssoftware/ccsr/dxccsr.jsp. Accessed August 4, 2021.

[24] Consumer Price Index. Medical Care, Series ID: CUUR0000SAM. https://data.bls.gov/cgi-bin/surveymost?cu.

[25] Kastelik JA, Thompson RH, Aziz I, Ojoo JC, Redington AE, Morice AH (2002). Sex-related differences in cough reflex sensitivity in patients with chronic cough. Am J Respir Crit Care Med..

[26] Morice AH, Kastelik JA (2003). Cough. 1: Chronic cough in adults. Thorax..

[27] Chung KF (2011). Chronic 'cough hypersensitivity syndrome': a more precise label for chronic cough. Pulm Pharmacol Ther.

[28] Matsumoto H, Tabuena RP, Niimi A (2012). Cough triggers and their pathophysiology in patients with prolonged or chronic cough. Allergol Int.

[29] Morice AH (2013). Chronic cough hypersensitivity syndrome. Cough.

[30] Morice AH, Faruqi S, Wright CE, Thompson R, Bland JM (2011). Cough hypersensitivity syndrome: a distinct clinical entity. Lung.

[31] Morice AH, Millqvist E, Belvisi MG (2014). Expert opinion on the cough hypersensitivity syndrome in respiratory medicine. Eur Respir J.

[32] Song WJ, Morice AH (2017). Cough hypersensitivity syndrome: a few more steps forward. Allergy Asthma Immunol Res.

[33] Kaplan AG (2019). Chronic cough in adults: make the diagnosis and make a difference. Pulm Ther.

[34] Palombini BC, Villanova CA, Araujo E (1999). A pathogenic triad in chronic cough: asthma, postnasal drip syndrome, and gastroesophageal reflux disease. Chest.

[35] Oppenheimer JJ, Meltzer EO, Bernstein JA, et al. The chronic cough experience in the United States: a patient survey (in preparation).

[36] McGarvey LP, Heaney LG, MacMahon J (1998). A retrospective survey of diagnosis and management of patients presenting with chronic cough to a general chest clinic. Int J Clin Pract.

