# Supplementary material for: Development of a natural language processing algorithm to detect chronic cough in electronic health records
Source: BMC Pulm Med. 2022 Jun 28;22:256. doi: 10.1186/s12890-022-02035-6 (PMC9238070; doi:10.1186/s12890-022-02035-6)
Supplement: Supplementary file 1 — Additional file 1: Supplemental Table 1. Examples of positive cough mentions in provider notes. Supplemental Table 2. Examples of negative and other non-applicable cough mentions. Supplemental Figure 1. Participant identification and observation periods. Supplemental Figure 2. Participant disposition diagram. [file 12890_2022_2035_MOESM1_ESM.docx]

## Appendices

Supplemental Table 1. Examples of positive cough mentions in provider notes

| Example text |
| --- |
| ‘Presents with *cough*’ |
| ‘Reports dry *cough* x 3 days’ |
| ‘Assessment: Subacute *cough* unresponsive to antibiotics’ |
| ‘Respiratory: *cough* but no shortness of breath’ |
| ‘Three-week history of *cough’* |
| ‘Patient reports chronic *coughing*’ |
| ‘*Coughing* history of four days’ |
| ‘Post-viral *tussis*’ |
| ‘Patient *coughed* for days’ |
| ‘He is a chronic *cougher*’ |
| ‘Patient reports he *coughs* all the time’ |

Text in *italics* indicates linguistic terms for cough.

Supplemental Table 2. Examples of negative and other non-applicable cough mentions

| Description / rationale | Example text |
| --- | --- |
| Negative statements | ‘*Negative* for cough and shortness of breath’ |
|  | ‘Patient *denies* cough and SOB’ |
|  | *‘No* cough reported’ |
|  |  |
| Hypothetical statements | ‘Keep cardiac pillow with him at all times, *in case of* coughing’ |
|  | ‘*If* cough occurs, take over the counter cough medication’ |
|  |  |
| Hedged statements | ‘Coughing *might* occur as he progresses’ |
|  | ‘patient *may* cough as his flu test is positive’ |
|  | ‘cough *may* occur’ |
|  |  |
| Not relevant to patient / general statements / family history / general instructions | ‘This medication is used for wheezing or coughing’ |
|  | ‘*Family history* of cough’ |
|  | ‘Cover mouth and nose with tissue when coughing’ |
|  |  |
| Not a relevant cough context (hemoptysis) | ‘Patient coughed up *blood*’ |
|  | ‘Still having persistent episodes of *hemoptysis*’ |

Text in *italics* represents statements that reflect a non-cough or non-applicable cough mention.

Supplemental Figure 1. Participant identification and observation periods


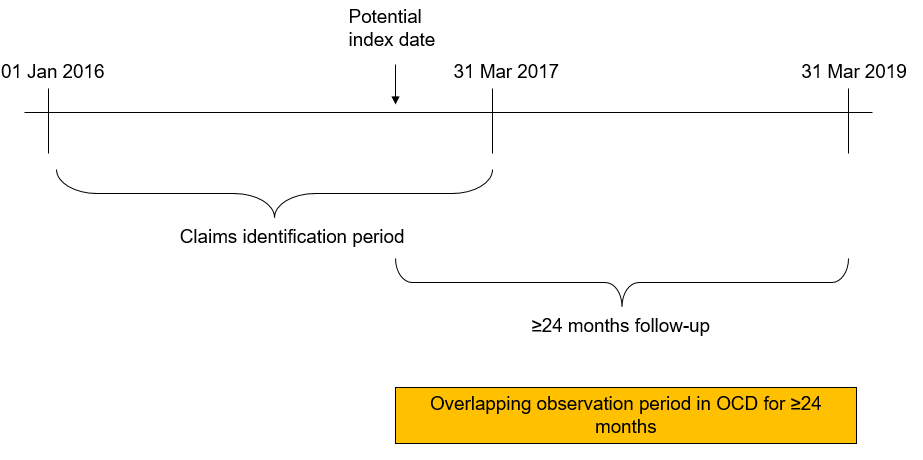


Potential participants were first identified in the Optum Research Database. The earliest observed date of enrollment for each participant within the identification period (January 1, 2016 to March 31, 2017) was set as the index date. Eligible participants had ≥24 months of continuous health plan enrollment after or including the index date, with overlapping observable data in both the Optum Research Database and the Optum Research Clinical Database during this observation period.

Supplemental Figure 2. Participant disposition diagram

ACE, angiotensin-converting enzyme
